# Sugar loss and enzyme inhibition due to oligosaccharide accumulation during high solids-loading enzymatic hydrolysis

**DOI:** 10.1186/s13068-015-0378-9

**Published:** 2015-11-26

**Authors:** Saisi Xue, Nirmal Uppugundla, Michael J. Bowman, David Cavalier, Leonardo Da Costa Sousa, Bruce. E Dale, Venkatesh Balan

**Affiliations:** DOE Great Lakes Bioenergy Research Center, Biomass Conversion Research Lab (BCRL), Chemical Engineering and Materials Science, Michigan State University, 3815 Technology Boulevard, Lansing, MI 48910 USA; USDA, Agricultural Research Service, National Center for Agricultural Utilization Research, Bioenergy Research Unit, Peoria, IL 61604 USA; DOE Plant Research Laboratory, Michigan State University, East Lansing, MI 48824 USA

**Keywords:** Recalcitrant oligosaccharides, High solids-loading, Commercial enzymes, AFEX-CS hydrolysate, Charcoal fractionation, Size exclusion chromatography, Enzyme inhibition

## Abstract

**Background:**

Accumulation of recalcitrant oligosaccharides during high-solids loading enzymatic hydrolysis of cellulosic biomass reduces biofuel yields and increases processing costs for a cellulosic biorefinery. Recalcitrant oligosaccharides in AFEX-pretreated corn stover hydrolysate accumulate to the extent of about 18–25 % of the total soluble sugars in the hydrolysate and 12–18 % of the total polysaccharides in the inlet biomass (untreated), equivalent to a yield loss of about 7–9 kg of monomeric sugars per 100 kg of inlet dry biomass (untreated). These oligosaccharides represent a yield loss and also inhibit commercial hydrolytic enzymes, with both being serious bottlenecks for economical biofuel production from cellulosic biomass. Very little is understood about the nature of these oligomers and why they are recalcitrant to commercial enzymes. This work presents a robust method for separating recalcitrant oligosaccharides from high solid loading hydrolysate in gramme quantities. Composition analysis, recalcitrance study and enzyme inhibition study were performed to understand their chemical nature.

**Results:**

Oligosaccharide accumulation occurs during high solid loading enzymatic hydrolysis of corn stover (CS) irrespective of using different pretreated corn stover (dilute acid: DA, ionic liquids: IL, and ammonia fibre expansion: AFEX). The methodology for large-scale separation of recalcitrant oligosaccharides from 25 % solids-loading AFEX-corn stover hydrolysate using charcoal fractionation and size exclusion chromatography is reported for the first time. Oligosaccharides with higher degree of polymerization (DP) were recalcitrant towards commercial enzyme mixtures [Ctec2, Htec2 and Multifect pectinase (MP)] compared to lower DP oligosaccharides. Enzyme inhibition studies using processed substrates (Avicel and xylan) showed that low DP oligosaccharides also inhibit commercial enzymes. Addition of monomeric sugars to oligosaccharides increases the inhibitory effects of oligosaccharides on commercial enzymes.

**Conclusion:**

The carbohydrate composition of the recalcitrant oligosaccharides, ratios of different DP oligomers and their distribution profiles were determined. Recalcitrance and enzyme inhibition studies help determine whether the commercial enzyme mixtures lack the enzyme activities required to completely de-polymerize the plant cell wall. Such studies clarify the reasons for oligosaccharide accumulation and contribute to strategies by which oligosaccharides can be converted into fermentable sugars and provide higher biofuel yields with less enzyme.

## Background

### Lignocellulosic biomass conversion

Concerns about energy security and environmental problems due to petroleum consumption provide an impetus to transition from the current fossil fuel system to a more sustainable energy system [1]. Nonfood plant biomass or cellulosic biomass, including agricultural residues [e.g., corn stover (CS), wheat straw], perennial grasses (e.g., switchgrass, miscanthus), forestry residues, herbaceous and woody crops, is the most abundant source that could be used as potential feedstock for producing renewable liquid fuels [2]. Since cellulosic biomass is likely to play an important role in future energy portfolios, numerous research efforts are underway globally to economically produce biofuels.

Nevertheless, several major issues impede the successful commercialization of cellulosic biomass conversion to liquid fuels [3]. These barriers include cost of biomass degrading enzymes, incomplete conversion of biomass to fermentable sugars and low ethanol productivity [4–6]. Cellulose and hemicelluloses in the plant cell wall are embedded in a complex matrix with lignin that make biomass highly recalcitrant [7]. Thus, numerous pretreatment technologies have been developed to overcome biomass recalcitrance, such as: hot water, steam explosion, lime, phosphoric acid, dilute acid (DA), ammonia [such as, ammonia fibre expansion (AFEX™), soaking in aqueous ammonia (SAA) and ammonia recycled percolation (ARP)] and ionic liquid (IL)-based pretreatments [8, 9]. DA, IL and AFEX pretreatments are of the most leading technologies and core programmes studied at the three US Department of Energy (DOE) funded bioenergy research centres, namely Great Lakes Bioenergy Research Center (GLBRC), Joint Bioenergy Institute (JBEI) and the BioEnergy Science Center (BESC). Considerable research has been done to streamline these processes to make them cost effective. In previous work, comparative studies of commercial enzyme mixture optimization and ethanol production of DA, IL and AFEX™ pretreated corn stover have been performed to improve downstream processing conditions and to understand factors contributing to the glucose and xylose yields during enzymatic hydrolysis [10, 11]. To reduce the cost in cellulosic ethanol production, high sugar concentrations are required to achieve high ethanol titers and to reduce the energy required to distill the ethanol from fermentation broths [12, 13]. However, as the solids loading increases, the viscosity of the hydrolysate also increases, contributing to mixing and mass transfer problems that reduce sugar conversion [14]. High solids loadings also leads to unproductive binding of enzyme to substrate [15] and product inhibition [16, 17], which are stumbling blocks for converting biomass to high concentrations of fermentable sugars.

Oligosaccharides are generated by incomplete digestion of glucan or xylan sugar polymers by water-soluble endo- and exo- enzymes. Most oligosaccharides are ultimately converted to glucose or xylose by β-glucosidase and β-xylosidase [18, 19]. However, some oligomeric sugars are not converted and thus accumulate in the hydrolysate. Oligosaccharides are produced by AFEX pretreatment and during high solid loading enzymatic hydrolysis [8, 20]. Gluco-oligosaccharides (DP 2–24) were found in AFEX corn stover (ACS) water extracts [21]. When presoaked wheat straw was treated with pressurised water at 195 °C for 12 min at a water-straw ratio of 5:1, most hemicelluloses were solubilized into water-soluble oligosaccharides as a mixture of xylo-oligomers and gluco-oligomers with DP ranging from 7–16 [22]. The soluble oligosaccharides present in switchgrass hydrolysates produced using commercial enzyme cocktails were a combination of both linear (gluco-) and branched (xylo-) oligomers, with DP ranging from 2–6 [23]. These unconverted oligosaccharides in hydrolysate reduce biofuel yields because most industrial ethanol-producing strains, including yeast and bacteria, consume only monomeric sugars (glucose and xylose) and do not have metabolic machinery to utilise oligomeric sugars [24–27].

### Mechanistic understanding of oligosaccharide accumulation

The exact reasons for oligosaccharide build-up in high-solids loading (HSL) hydrolysis are unknown, but there are several possible mechanisms to explain this phenomenon. Monomeric sugars (glucose, xylose, mannose, galactose, etc.) and cellobiose have been determined to exert strong end-product inhibition on cellulase and β-glucosidase [28, 29], and oligomeric sugars (xylo-oligomers and gluco-oligomers) can inhibit cellulases during hydrolysis even more strongly [22, 30, 31]. Accumulation of xylo-oligomers reduces ethanol yields by inhibiting cellulase enzymes, especially CBH I and CBH II [32, 33], thereby reducing cellulose hydrolysis to glucose. Competitive inhibition of cellulases by high DP xylo-oligomers is greater than other end products (glucose, xylose) [30]. Oligosaccharides with a DP ranging from 7 to 16 resulting from wheat straw hydrothermal pretreatment are approximately 100-fold stronger inhibitors of *Trichoderma reesei* CBHs than cellobiose [22]. A possible explanation was proposed by the author that the xylo-oligomers and gluco-oligomers may mimic the structure of the cellulose chain and bind to more glucose unit binding sites in the active site tunnel than cellobiose. Alternatively, the presence of side-chain substituents on arabinoxylan, including acetyl, arabinofuranosyl and glucopyranosyl uronic acid, may hinder the formation of enzyme-substrate complexes, and thus impede enzymatic hydrolysis [34]. Current commercial enzyme cocktails require accessory enzymes that can cleave these linkages [35]. Such accessory enzymes including glucuronidases, β-xylosidases, α-l-arabinofuranosidases and acetyl esterases are essential in achieving complete degradation of heteroxylans [18, 34, 36–39].

### Previous accomplishments on oligosaccharide purification and characterization

Other studies have explored oligosaccharide recalcitrance, including the purification and/or characterization of oligosaccharides [37, 38, 40–43]. Neutral gluco-oligosaccharides in ACS water extracts were enriched by solid-phase extraction (SPE), followed by high-performance liquid chromatography (HPLC) separation and electrospray ionisation time-of-flight mass spectrometry (ESI-TOF-MS) [21]. High-purity xylo-oligosaccharide fractions with DP ranging from 2 to 14 were isolated from hydrothermal pretreatment hydrolysate of birchwood xylan by gel permeation chromatography [44]. For structural elucidation on the chromatographic time scale, nonselective multiplexed collision-induced dissociation was performed for quasi-simultaneous acquisition of oligosaccharide molecular and fragment masses in a single analysis [21]. Hydrophilic interaction liquid chromatography mass spectrometry/mass spectrometry (HILIC–MS/MS) was successfully used to characterise reducing end-labelled xylo-oligosaccharides [41]. Feruloylated xylo-oligomers from thermochemically treated corn fibre were pooled and fractionated by a solid-phase C-18 column and a Bio-Rad P2 gel column and further purified with reverse-phase high-performance liquid chromatography (RP-HPLC). Electrospray ionisation mass spectrometry (ESI-MS^n^) and nuclear magnetic resonance (NMR) were then used for structure elucidation. Interestingly, several oligosaccharide analogues contained an α-l-galactopyranosyl-(1-2)-β-d-xylopyranosyl-(1-2)-5-*O*-trans-feruloyl-l-arabinofuranose side chain attached to the *O*-3 position of a xylose comprising the β-1-4 linked backbone [45]. Arabino-xylooligomers derived from switchgrass xylan were characterised by RP-HPLC-MS^n^ [37]. Side chain substitutions of (1-2)-β-xylose-(1-3)-α-arabinose were identified from the products of swichgrass xylan hydrolysis using commercial enzymes with supplementation of α-arabinofuranosidase, indicating that required activity for this linkage is lacking from commercial enzyme preparations [43]. With all these research efforts, however, only a few studies have focused on the nature of oligosaccharide buildup during HSL hydrolysis in terms of release of oligomeric sugars, changes in the oligomer chain length during the course of hydrolysis, ratios of different DP oligomers and their distribution profiles [21, 23, 46]. We present here the first methodology for large-scale purification of recalcitrant oligosaccharides from AFEX treated corn stover hydrolysate (ACSH) under high solids loading. Composition analysis, recalcitrance studies, and investigating enzyme inhibition by recalcitrant oligosaccharide on commercial enzyme cocktail have helped us define the characteristics of the mechanism of oligosaccharide accumulation.

## Results and discussion

### Oligosaccharide accumulation

**Fig. 1:**
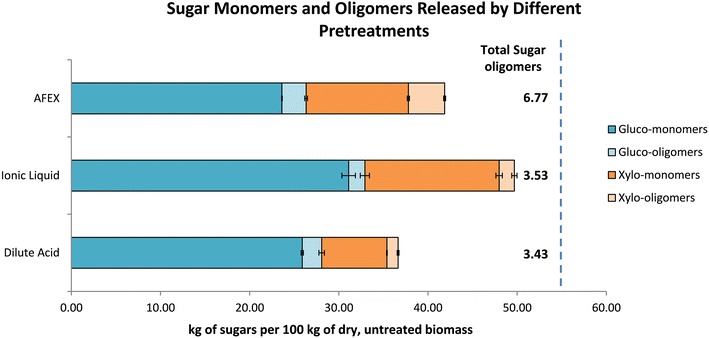
Sugar monomers and oligomers released by different pretreatments [dilute acid (DA), ionic liquid (IL), ammonia fibre expansion (AFEX)]. Enzymatic hydrolysis conditions: 6 % glucan loading, 72 h hydrolysis, 15 mg/g enzyme loading (Ctec2:Htec2:MP—1:1:1)

Glucan and xylan conversion of CS using three different leading pretreatment technologies (dilute acid: DA, ionic liquids: IL, ammonia fibre expansion: AFEX) under commercial enzymes treatment were tested to see if oligosaccharide accumulation is a universal phenomenon or pertains only to particular pretreatments (Fig. 1). The optimal commercial enzyme combinations (Ctec2:Htec2:Multifect Pectinase) for different pretreated biomass was shown to be different in our previous work [10]. Nevertheless, to reduce factors that might affect the sugar conversion and have a comparative results between three different pretreatment methods, we chose 1:1:1 (percentage total protein loading basis) for our experiments. Regardless of the pretreatment method used, 3.4-6.1 % of sugars (glucose and xylose) in dry untreated corn stover accumulated as oligomers. These recalcitrant oligosaccharides are not converted to fermentable sugars and thereby reduce biofuel yields. The oligosaccharides also inhibit both fermentation rate and biomass hydrolysis as stated above. Among these three pretreatment technologies (IL, DA and AFEX), AFEX™^1^ is a thermochemical biomass pretreatment that cleaves the lignin–carbohydrate complex (LCC) linkages and improves the digestibility of biomass by relocating lignin and creating pores [47, 48]. It is a dry-to-dry process without any washing step, and no exogenous nutrition supplementation is needed for downstream fermentation. Nevertheless, highest contents of oligosaccharides (gluco-oligomers and xylo-oligomers) were produced during AFEX pretreatment (Fig. 1). Therefore, rather that handling different pretreated biomass, we decided to choose AFEX pretreated corn stover to broadly represent alkaline pretreatment process and try to have more in-depth understanding of recalcitrant oligosaccharides produced during enzyme hydrolysis. We chose to use ACSH produced under high solids-loading (25 %) enzymatic hydrolysis to study the accumulation profile of recalcitrant oligosaccharides and their structure characterization.

**Fig. 2:**
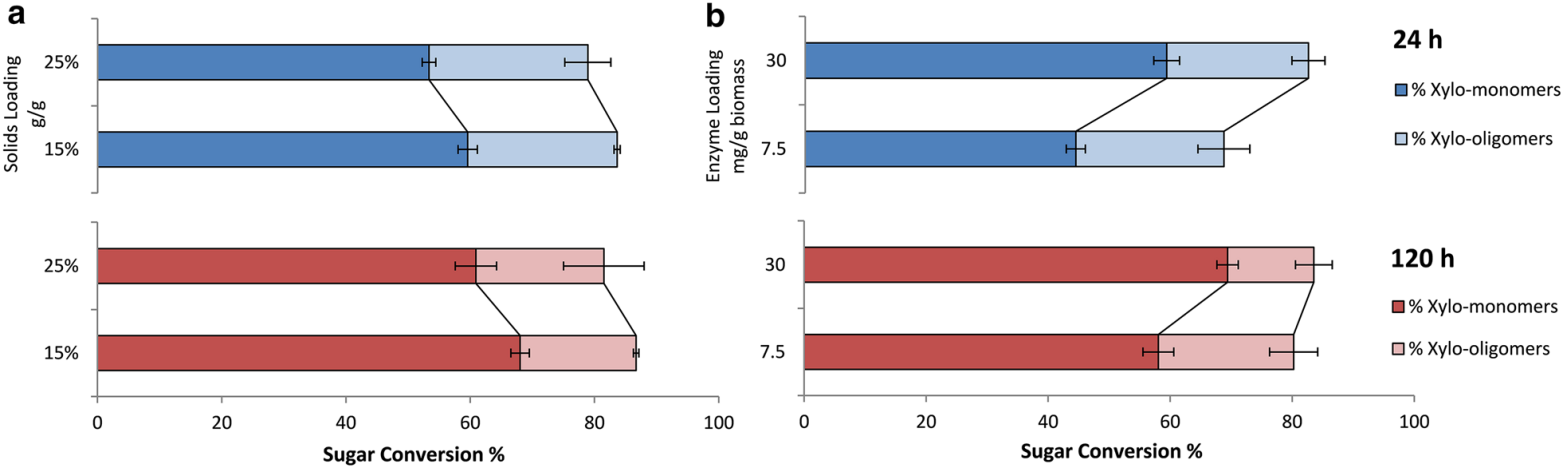
Effect of solids loading and enzyme loading on xylo-oligomers accumulation in ACS enzymatic hydrolysis. **a** Enzyme loading was fixed at 18.75 mg/g glucan (optimised mixture, Ctec2:Htec2:MP—1:1:1). Two *solids* loadings (15 and 25 %) were compared at 24 and 120 h; **b** *solids* loading was fixed at 20 %. The effects of two enzyme loadings (7.5 and 30 mg/g glucan, Ctec2: Htec2: MP—1:1:1) were compared at 24 and 120 h

As more than 25 % of the initial xylan content remains as xylo-oligomers after enzymatic hydrolysis of ACS while gluco-oligomers accumulate (<10 %) to a lesser extent, different solids loadings (Fig. 2a) and enzyme loadings (Fig. 2b) were tested for their effects on xylo-oligomer accumulation. Here solids loading (g/g) is defined by the amount of dry material that enters the process divided by the total mass of material and water added to the material [49], and enzyme loading (mg/g) is defined as the amount of protein added in the process divided by the amount of glucan in pretreated biomass. Sugar conversion is defined as the amount of xylose or xylo-oligomers produced after enzymatic hydrolysis divided by the amount of xylan in ACS (AFEX-pretreated corn stover). In Fig. 2a, approximately 80 % of total xylan conversion was achieved in the first 24 h at both 15 and 25 % solids loadings with 18.75 mg/g enzyme loading. This value does not change significantly after 120 h of enzymatic hydrolysis. The monomeric xylose content increased by 7–10 % for both solids loadings when the hydrolysis time was extended from 24 to 120 h. For 15 % solids loading, the xylo-oligomers content decreased from 24 to 18 % after 120 h hydrolysis. At 25 % solid loading, xylo-oligomers content decreased from 28 to 24 % after 120 h hydrolysis, indicating that more xylo-oligomers accumulated in high solids-loading enzymatic hydrolysis. The effect of enzyme loadings at a fixed solid loading (20 %) was also tested (Fig. 2b). At 20 % solids loading, enzymatic hydrolysis with 7.5 mg/g enzyme loading achieved 70 % xylan conversion, while 30 mg/g enzyme loading achieved over 80 % in the first 24 h. At 120 h, 7.5 mg/g of enzyme achieved nearly the same overall conversion (80 %) as did under the higher enzyme loading (83 %). However, higher level of xylo-oligomers (22 %) is still present at 7.5 mg/g enzyme loading than at 30 mg/g enzyme loading (13 %). As more xylo-oligomers accumulate at the high solids loading, we chose 25 % solids loading (7.94 % glucan loading) ACSH to produce oligosaccharides in a larger scale.

### Methodology and mass balance for large-scale recalcitrant oligosaccharide production

**Fig. 3:**
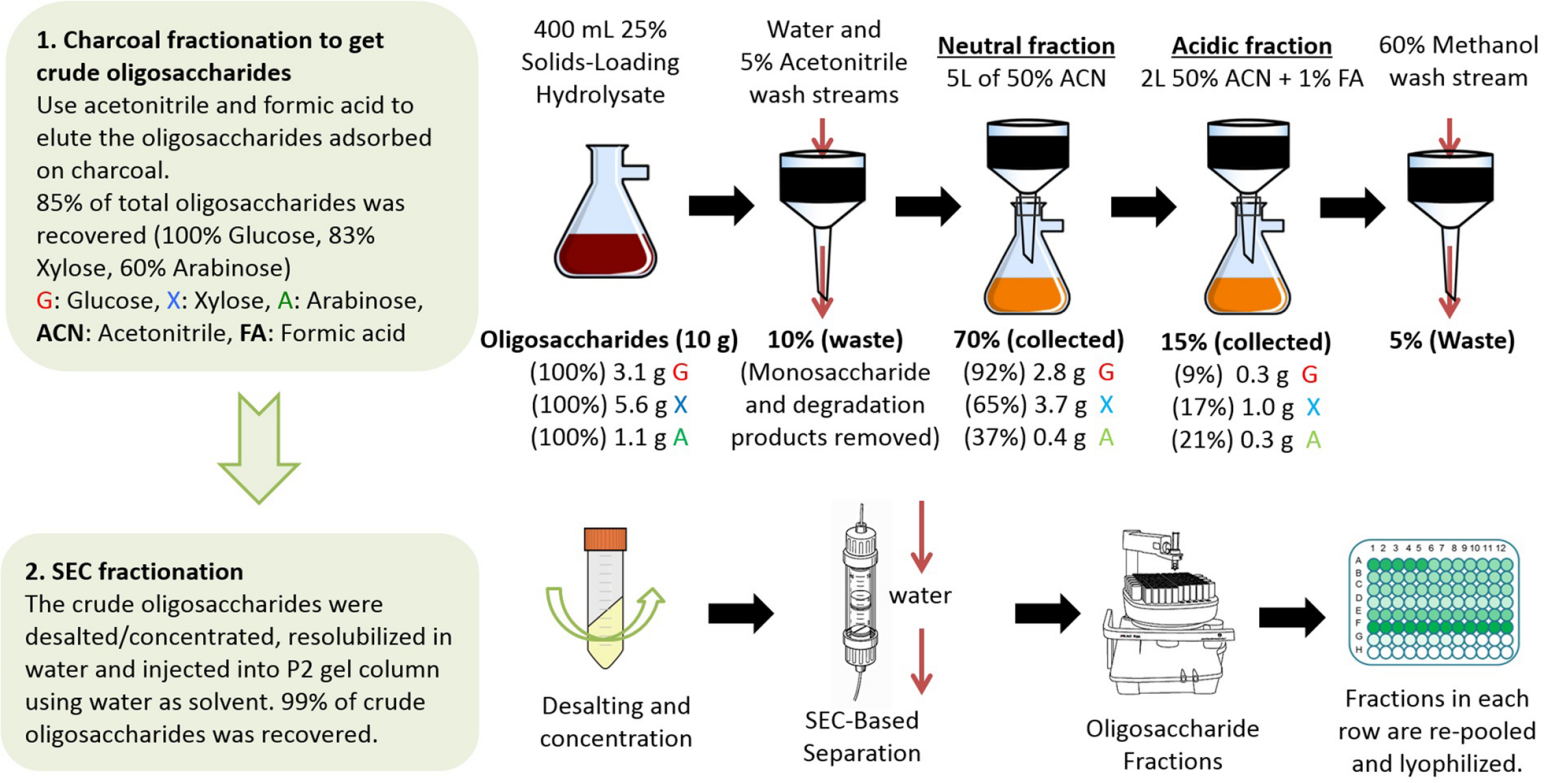
Mass balance and methodology of oligosaccharides yield during charcoal fractionation. Oligosaccharides were separated from ACSH by activated charcoal bed and further fractionated by size exclusion chromatography (SEC). Dilute sulfuric acid hydrolysis was performed for sugar composition and mass balance. *G* glucose; *X* xylose; *A* arabinose, *ACN* acetonitrile, *FA* formic acid

To better understand these oligosaccharides, the first step is to separate them from the hydrolysate by removing monomeric sugars, proteins, salts and other lignin degradation products present (Fig. 3). High solids-loading (25 %, i.e., ~7.9 % glucan loading) ACS hydrolysate was prepared for large-scale separation of oligosaccharides. Enzymatic hydrolysis of ACS was performed using a commercial enzymes mixture including Ctec2, Htec2 and Multifect Pectinase (20 μg protein/mg glucan, 2:1:1 ratio). After 96 h hydrolysis, the hydrolysate was centrifuged, filtered and stored at 4 °C prior to charcoal fractionation. Oligosaccharide analyses were performed to determine the composition of glucose, xylose and arabinose. The composition of sugar monomers and oligomers in 25 % solids loading ACSH is shown in Table 1. The soluble sugar percentage is the weight percentage of oligomers in each individual sugar (monomers plus oligomers), and the biomass percentage is the weight percentage of oligosaccharides based on starting material (ACS).

**Table 1:** Concentration of sugar monomers and oligomers in 25 % solids loading ACS hydrolysate and inlet untreated biomass

	Glucose	Xylose	Arabinose	Total sugar
Monomers (g/L)	77	43	6	*126*
Oligomers (g/L)	8	14	3	*25*
Oligo percentage in soluble sugar	9 %	25 %	33 %	*17* *%*
Oligo percentage in untreated biomass	2 %	4 %	1 %	*7* *%*

Oligo percentage in soluble sugar is based on individual sugar (glucose, xylose and arabinose), while the oligo percentage in total sugar is based on the sum of all three sugars

Italic values indicate the concentration/percentage of total sugars (glucose, xylose and arabinose) in ACS hydrolysate or inlet untreated biomass

An activated charcoal and Celite column was used to adsorb oligosaccharides from 25 % solids loading ACS hydrolysate [50, 51]. After fully adsorption of oligosaccharides, most of neutral oligosaccharides were eluted from the charcoal/Celite column using 50 % acetonitrile (v/w), followed by desorption of more acidic oligosaccharides using 50 % acetonitrile containing 1 % formic acid (v/w). The neutral and acidic oligosaccharide elution streams were collected as crude oligosaccharide fractions and were processed for further fractionation by size exclusion chromatography (SEC).

Crude oligosaccharides were concentrated to remove the organic solvent and additives prior to SEC fractionation. The concentrated fractions were re-suspended in water (50 mL of neutral fractions/55 mL of formic acid fractions) and used for sugar compositional analysis by acid hydrolysis, recalcitrance studies and SEC fractionation. A mass balance for oligosaccharides separated in the process and sugar recovery were calculated based on these acid hydrolysis results.

**Fig. 4:**
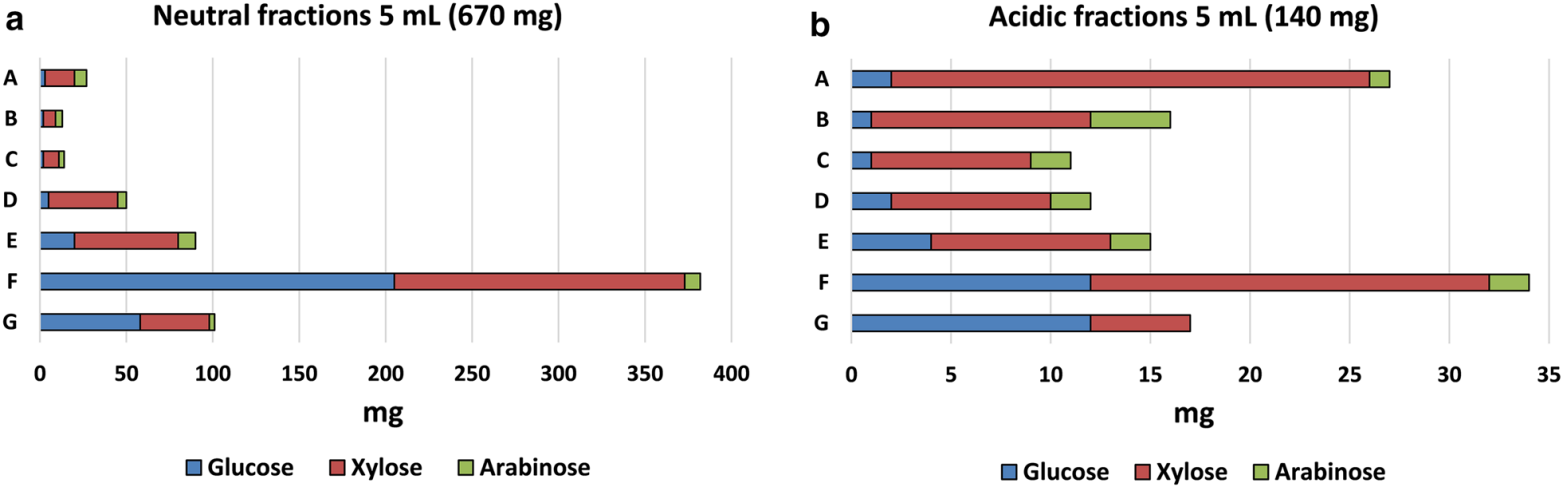
Mass distribution profile and monomeric compositions of oligosaccharide fractions after SEC separation. 5 mL of concentrated neutral fractions (containing 670 mg oligosaccharides) and acidic fractions (containing 140 mg oligosaccharides) were injected into the SEC column. Fractions were re-pooled from *row A–G* and lyophilized. *G* glucose; *X* xylose; *A* arabinose

The concentrated neutral and acidic fractions were further fractionated based on their molecular weight using SEC. 5 mL of concentrated neutral fractions (containing 670 mg oligosaccharides) and acidic fractions (containing 140 mg oligosaccharides) was injected into the SEC column separately. 90 fractions (A1-12, B1-12, C1-12, D1-12, E1-12, F1-12, G1-12, H1-6, 10 mL in each tube) were collected from each run and characterised using acid hydrolysis and enzyme digestion assays. Fractions in the same row were re-pooled and lyophilized to produce dry samples A–G (H fractions are predominantly monomers according to composition analysis). Mass distribution of a total of 14 pooled samples was generated (Fig. 4, row A–G for both neutral and acidic oligosaccharides). The composition of oligosaccharides separated using SEC fractionations were also analysed in the same way as crude oligosaccharides.

A complete mass balance was carried out following the charcoal fractionation process (Figs. 3, 4). Using this oligosaccharide separation process we were able to separate 400 mL of 25 % solids loading hydrolysate containing about 10 g of mixed oligosaccharides (in accordance with the sugar concentration in Table 1) in one activated charcoal bed. About 15 % (1.5 g) of 10 g oligosaccharides in the hydrolysate were lost due to co-elution with monomeric sugars and irreversible binding to the charcoal matrix. About 85 % (8.5 g) of oligosaccharides were recovered containing 6.9 g neutral oligosaccharides and 1.7 g acidic oligosaccharides. Acid hydrolysis showed that acidic oligosaccharides had higher xylose content (63 vs. 54 %) and arabinose content (19 vs. 6 %) than neutral oligosaccharides. After SEC fractionation, more than 99 % of these crude oligosaccharides were recovered. The mass distribution profile in Fig. 4 shows that most neutral fractions were enriched in low DP oligosaccharides (row F and G) while the acidic fractions were distributed more evenly in high and low DP (row A and F) oligosaccharides. Acid hydrolysis revealed that high DP oligosaccharides (A, B, C rows) contain more xylose than low DP ones (F, G rows) for both neutral and acidic fractions.

### Recalcitrance of crude and SEC-fractioned oligosaccharides towards commercial enzymes

**Fig. 5:**
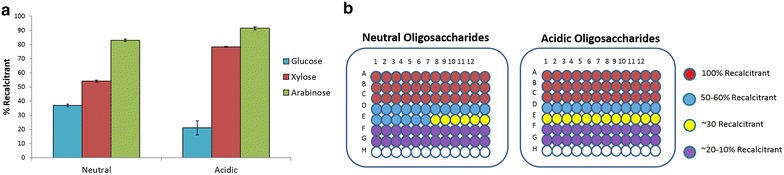
Recalcitrance study of neutral and acidic oligosaccharides. **a** Recalcitrance of crude oligosaccharides after charcoal fractionation based on monomeric sugar composition; **b** recalcitrance of oligosaccharides fractions separated by SEC determined. Enzymatic hydrolysis was performed in 0.5 mL reaction volumes with commercial enzymes (Ctec2:Htec2:MP) in 1:1:1 ratio for 24 h hydrolysis at 50 °C and pH 4.8. Maximum sugar concentration per well was 3 g/L while the minimum was 0.2 g/L (determined by acid hydrolysis). Enzyme loading is 60 μg/well

The oligosaccharides extracted from the initial hydrolysate (8.5 g) by charcoal fractionation were further fractionated by SEC chromatography. A series of hydrolysis using commercial enzyme mixtures (Ctec2 + Htec2 + MP) were performed on both charcoal fractionated oligosaccharides and SEC-fractionated oligosaccharides to determine the level of recalcitrance in each fraction (0.5 mL reaction volumes with commercial enzymes (Ctec2:Htec2:MP) in 1:1:1 ratio for 24 h hydrolysis at 50 °C and pH 4.8 and 20 mg/g oligosaccharide enzyme loading). Here we define recalcitrance as the percentage of oligosaccharides not hydrolysed into monomeric sugars. Figure 5a shows that among the three monomeric sugars (glucose, xylose and arabinose), the gluco-oligomers were most digestible (20–40 % recalcitrance), followed by xylo-oligomers (50–80 % recalcitrance), and arabino-oligomers were found to be highly recalcitrant (85–95 %). Additionally, acidic oligosaccharides (70 % recalcitrant) were more recalcitrant than neutral oligosaccharides (49 % recalcitrance). The higher recalcitrance of acidic fractions towards neutral ones is consistent with a previous study that enrichment of certain substituents (arabinose, acetyl group, glucuronic acid and uronic acid) correlated with increasing recalcitrance characteristic in corn residues [34]. It is reasonable to believe that multiple substitutions on the arbinoxylan backbone, especially in acidic fraction, can hinder the accessibility of oligosaccharides to commercial enzyme mixtures, leading to accumulation of unhydrolysed oligosaccharides in hydrolysate.

Based on the sugar conversion of diluted SEC-fractionated oligosaccharides incubated with commercial enzyme mixtures, we were able to separate the crude oligosaccharides into three different groups (Fig. 5b): (1) highly recalcitrant (100 % recalcitrant, row A, B and C); (2) moderately recalcitrant (30–60 % recalcitrant, row D and E); and (3) less recalcitrant (10–20 % recalcitrant, row F and G). The highly recalcitrant oligosaccharides accounted for ~15 % (1.3 g) of the crude oligosaccharides separated from the hydrolysate. The remaining oligosaccharide fractions (7.2 g) were found to be partially digestible when subjected to commercial enzymes (Ctec2, Htec2 and MP). The recalcitrance results from Fig. 5b show that more than 80 % (w/w) of crude oligosaccharide mixtures can be partially digested after separation from monomeric sugars and being diluted to concentrations lower than 10 g/L. Oligosaccharide accumulation in the hydrolysate may result either from inhibition of glycosyl hydrolases by degradation products or oligosaccharides, or because of the high concentrations of monomeric sugars that are produced during enzyme hydrolysis.

### Inhibition of commercial enzymes by oligosaccharides

**Fig. 6:**
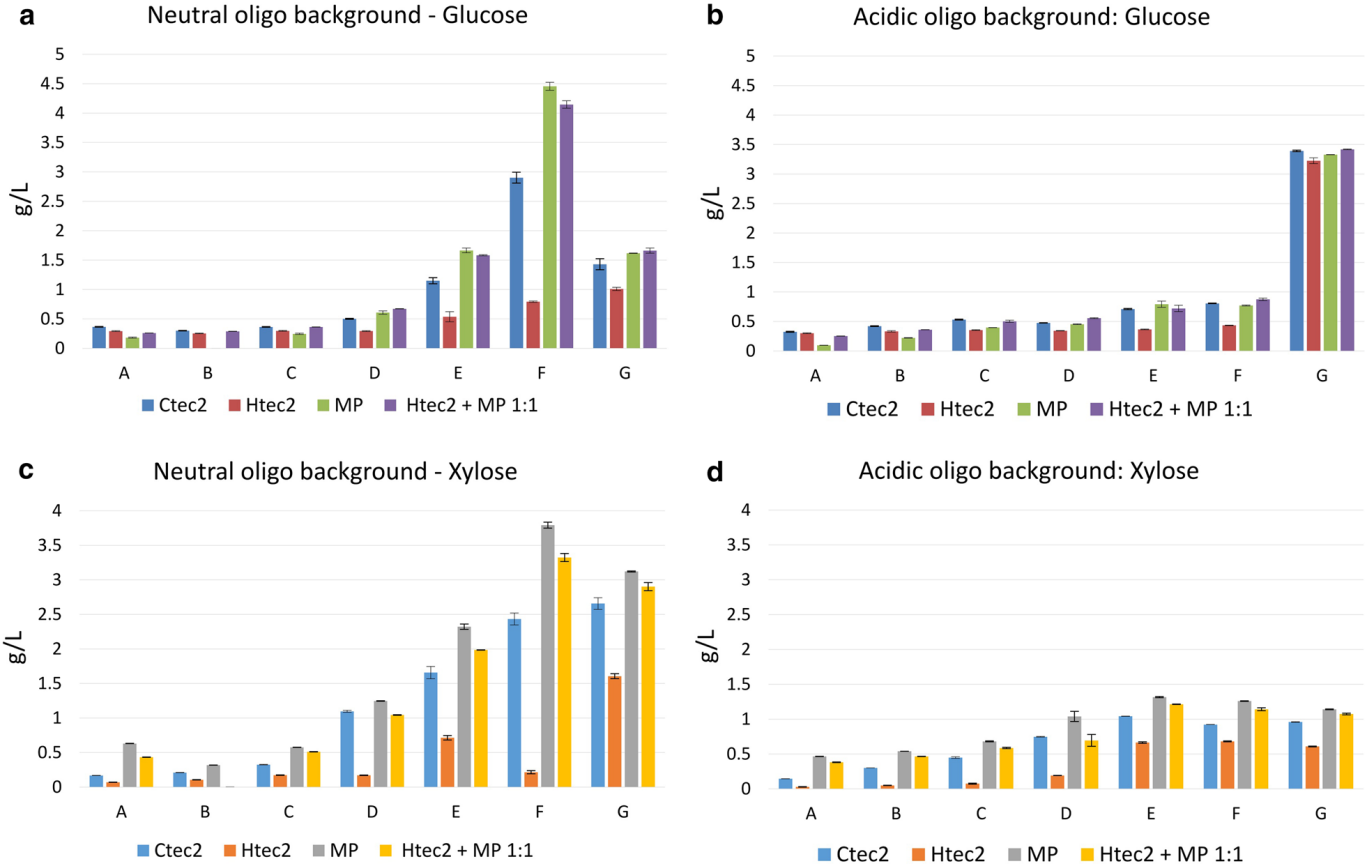
Glucose and xylose background for oligosaccharides under commercial enzyme mixtures. Clusters labelled with* A–G* represented different fractions after SEC separation. Each fraction was treated with four enzyme preparations (Ctec2, Htec2, MP and Htec2 + MP 1:1). Microplate enzymatic hydrolysis condition: Pure substrates (Avicel and beechwood xylan) were added to 1.25 % solids loading 500 μL reaction volume. Enzyme loading was 10 mg/g substrate with 24 h hydrolysis at 50 °C, 10 rpm and pH 4.8. **a**, **c** the glucose and xylose release of neutral oligosaccharides under commercial enzyme mixtures; **b**, **d** the glucose and xylose release of acidic oligosaccharides under commercial enzyme mixtures

**Fig. 7:**
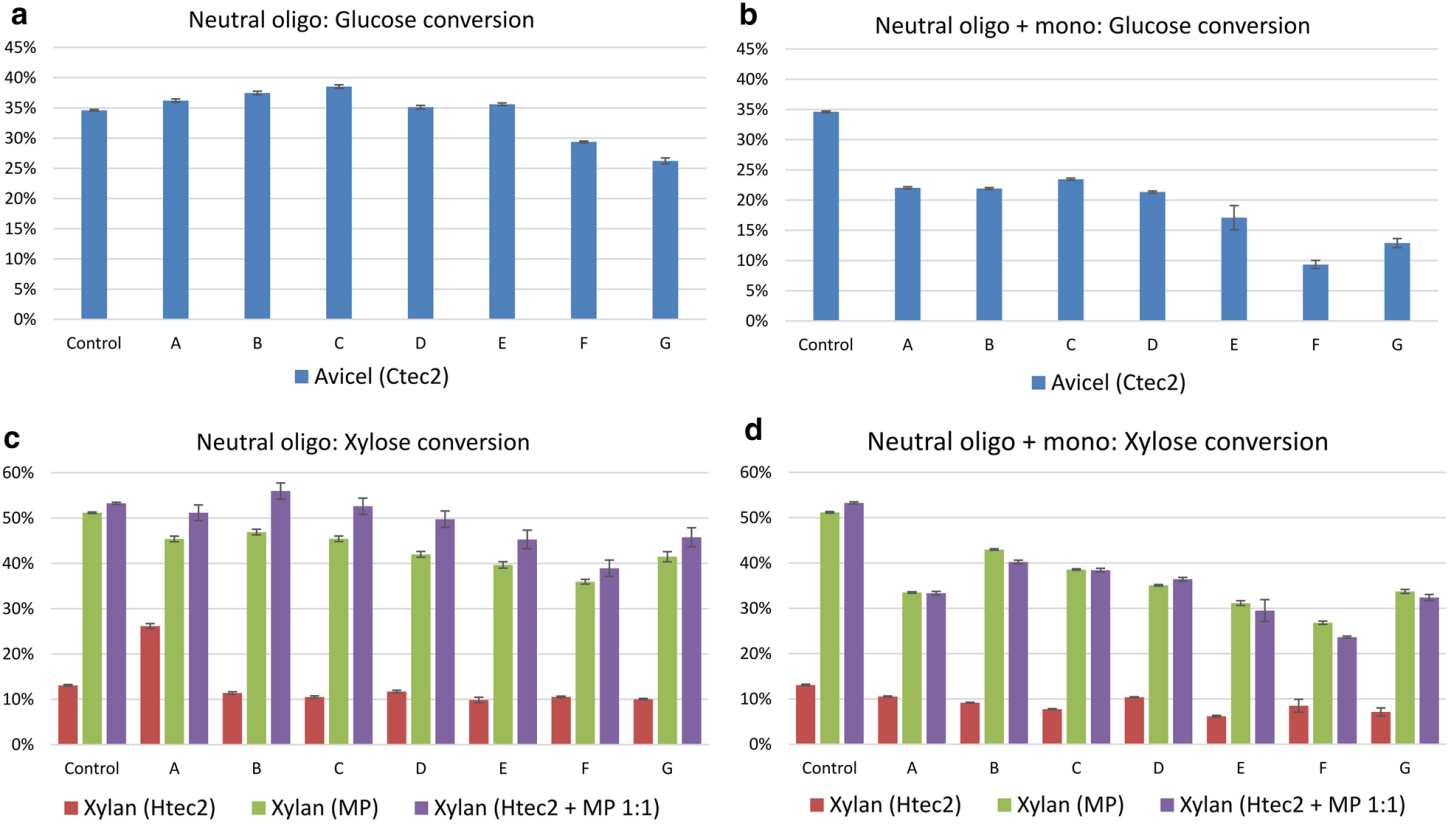
Neutral oligosaccharide inhibition on pure substrate enzymatic hydrolysis with or without monomeric sugar inhibition. Clusters labelled with *A–G* represented different pooled fractions after SEC separation. Avicel was treated with Ctec2 for glucose conversion; beechwood xylan was treated with Htec2, MP and Htec2 + MP 1:1 for xylose conversion. Microplate enzymatic hydrolysis condition: pure substrates (Avicel and beechwood xylan) were added to 1.25 % solids loading 500 μL reaction volume. Enzyme loading was 10 mg/g substrate with 24 h hydrolysis at 50 °C, 10 rpm and pH 4.8. Oligosaccharide concentration was at 10 g/L; monomeric sugars were at 20 g/L glucose, 10 g/L xylose. **a**, **c** the glucose and xylose conversion of substrates with neutral oligosaccharides inhibition; **b**, **d** the glucose and xylose conversion of substrates with neutral oligosaccharides inhibition and monomeric sugar inhibition

**Fig. 8:**
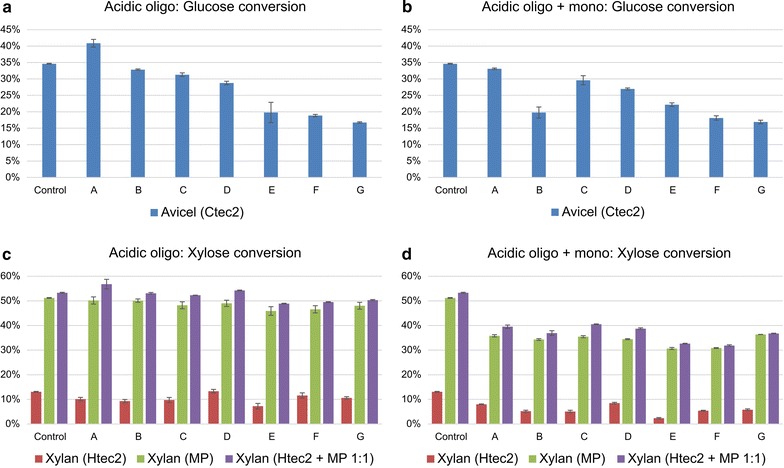
Acidic oligosaccharide inhibition on pure substrate enzymatic hydrolysis with or without monomeric sugar inhibition. Clusters labelled with* A–G* represented different fractions after SEC separation. Avicel was treated with Ctec2 for glucose conversion; beechwood xylan was treated with Htec2, MP and Htec2 + MP 1:1 for xylose conversion. Microplate enzymatic hydrolysis condition: pure substrates (Avicel and beechwood xylan) were added to 1.25 % solids loading 500 μL reaction volume. Enzyme loading was 10 mg/g substrate with 24 h hydrolysis at 50 °C, 10 rpm and pH 4.8. Oligosaccharide concentration was at 10 g/L; monomeric sugars were at 20 g/L glucose, 10 g/L xylose. **a**, **c** the glucose and xylose conversion of substrates with acidic oligosaccharides inhibition; **b**, **d** the glucose and xylose conversion of substrates with acidic oligosaccharides inhibition and monomeric sugar inhibition

It has been reported that oligosaccharides inhibit commercial enzyme mixtures [22, 29–32]. To study the inhibition on commercial enzymes by fractionated oligosaccharides with different degree of DPs and degree of recalcitrance, the activities of commercial enzymes on Avicel and beechwood xylan, with and without SEC fractionated acidic or neutral oligosaccharides, were measured with micro-plate assay (enzyme loading of 10 mg/g substrate, i.e., 62.5 μg enzyme per well, for 24 h hydrolysis at 50 °C, 10 rpm and pH 4.8, types and ratio of enzymes are stated below). Inhibition tests were performed with varying concentrations of crude oligosaccharides (0.5–10 g/L). The enzyme activities in the commercial enzyme preparations are different: Ctec2 is cellulases mixture blended with high level of β-glucosidases and some hemicellulases; Htec2 is a endoxylanase with cellulase background (Novozyme: http://bioenergy.novozymes.com/en/cellulosic-ethanol/advantages/cellic/Pages/default.aspx); Multifect Pectinase contains high levels of pectinase, mannanase and some β-glucosidase [52]. Four groups of inhibition experiments were performed, namely (1) Enzyme + Substrate (blank control); (2) Enzyme + Substrate + Oligosaccharides (to test the inhibition of oligosaccharides on enzymes); (3) Enzyme + Substrate + Oligosaccharides + Monomeric sugars (to test the inhibition of both oligosaccharides and monomeric sugars on enzymes); (4) Enzyme + Oligosaccharides (for background subtraction, Fig. 6). The sugar levels from Group 2 experiments were subtracted by the background oligosaccharide (Group 4) sugar level, and the levels from experiment Group 3 were subtracted by both the oligosaccharide background (Group 4, Fig. 6 ) and the supplemented monomeric sugar levels. The monomeric sugars produced (glucose, xylose and arabinose) in experimental Groups 2 and 3, after subtraction, were compared with control Group 1 (Figs. 7, 8). If the sugar conversion levels are lower than the control group, the oligosaccharide fraction is considered inhibitory to commercial enzymes.

We started with oligosaccharide concentration ranging from 0.1–2.5 g/L and no inhibition was observed on the enzymes; this is not surprising since the concentration of oligosaccharides in high-solids loading ACSH is approximately 20 g/L. Therefore, we tested the inhibitory effect of these oligosaccharides at higher concentrations (10 g/L). The enzyme preparations used for different substrates were also different. Avicel was hydrolysed using Ctec2 to test inhibition effect of oligosaccharides on glucose conversion with cellulases; beechwood xylan contains mixture of xylose and glucuronic acid and was treated with Htec2 and MP individually and then using enzyme mixture of Htec2 and MP in 1:1 mass ratio at 10 mg/g to test the inhibition of oligosaccharides on xylose conversion with xylanase, pectinase and other hemicellulases. Monomeric sugars (20 g/L glucose, 10 g/L xylose) based on 50–60 % conversion of xylan and glucan from CS were also added to the system to evaluate the role of monomeric sugars on enzyme inhibition.

The concentration of sugars released at the same oligosaccharide concentration (10 g/L) and the same enzyme dosage (10 mg/g) is a measure of the recalcitrance of the given oligosaccharide mixture (Fig. 6). The background concentrations of glucose and xylose in Fig. 6 were consistent with the recalcitrance study results showed in Fig. 5b. For both neutral and acidic fractions, high DP fractions (A, B and C) are more recalcitrant and low DP oligosaccharides (row E, F and G) could be partially digested to mono-saccharides after separation from crude oligosaccharides. We believe that neutral fraction G consists of mostly monomers and degradation product impurities, resulting in a much lower glucose release than fraction F. Arabinose conversion was not shown here because of its low content in these oligosaccharides and no conclusions can be drawn due to large standard deviation. For both glucose and xylose conversion, it was clearly shown that acidic fractions are more recalcitrant than the neutral fractions. The resulting glucose conversions of most high to medium DP fractions (row A–F) for acid fractions were less than 1 g/L, Only the lowest DP fraction G is partially convertible (~3.5 g/L) (Fig. 6b). Interestingly, Multifect Pectinase exhibited higher activity on glucose and xylose conversion than Ctec2 and Htec2. For example, when neutral fraction F was treated with MP, ~4.5 g/L glucose was released (4.1 g/L for Htec2 + MP 1:1 treatment) while only 2.9 g/L glucose was released when treated with Ctec2, and treatment with Htec2 gave the lowest sugar concentration of 0.8 g/L glucose (Fig. 6a). Same phenomena can be observed on the xylose release on both neutral and acidic fractions (Fig. 6c/d), indicating that Htec2 (mainly endoxylanases) lacks the enzyme activity to digest the unhydrolysed oligosaccharides.

Inhibitory effects from neutral oligosaccharide fractions (Fig. 7a/c) and from a combination of oligosaccharides and monomeric sugars (Fig. 7b/d) were further studied. The micro-plate activity assays were performed under similar condition to Fig. 6, with the only difference that both oligosaccharides and monomeric sugars were added to the reaction mixture containing pure substrates and enzymes to test their inhibitory effects. At least four important trends can be deduced from the sugar inhibition profiles given in Fig. 7: (1) low DP oligosaccharides were more inhibitory (especially fraction F) than high DP oligosaccharides on glucose and xylose conversion; (2) neutral oligosaccharides were more inhibitory toward xylose conversion (Fig. 7c) while their inhibitory effect on glucose conversion is not as significant. With neutral fraction F, the xylose conversion of xylan treated with MP is 50 % (13 % reduction from control) while the glucose conversion of Avicel treated with Ctec2 is 37 % (only 6 % reduction from control); (3) the activity of Htec2 on beechwood xylan is much lower compared with MP and their 1:1 mixtures (Fig. 7c/d), indicating that xylanases are made more effective in sugar conversion because of the action of accessory enzymes; (4) supplementing monomeric sugar into the hydrolysis solution led to stronger inhibition of both glucose conversion (15–20 % reduced) and xylose conversion (10–15 % reduced, comparing Fig. 7a/c with b/d).

The inhibitory effect of acidic oligosaccharides was different from those of neutral oligosaccharides (Fig. 8) in the following ways. Low DP oligosaccharides were more inhibitory to glucose conversion than high DP oligomers. With acidic fraction G, the glucose conversion of Avicel treated with Ctec2 was 20 % (23 % reduction from control, Fig. 8a), while xylose conversion of xylan under all three enzyme preparations with different DPs of oligosaccharides was similar to control group (~60 % with MP and ~15 % with Ctec2, Fig. 8c). The inhibitory effect of acidic oligosaccharides on xylose conversion increased with the addition of monomeric sugars (20 % reduction for MP and ~5 % reduction for Ctec2, Fig. 8c/d), but no significant conversion decrease was observed for glucose conversion (Fig. 8a/b). Overall, the inhibitory effect of both neutral oligosaccharides and acidic oligosaccharides was observed on commercial enzyme cocktails (Ctec2, Htec2 and MP). As both cellulases and hemicellulases in the commercial enzymes were inhibited by oligosaccharides, and neither the oligosaccharides nor pure substrates could be degraded completely, augmenting the missing enzyme activities lacking in commercial enzyme cocktails would help break down the recalcitrant linkages in the oligosaccharides. Integrated biological processes like simultaneous saccharification and co-fermentation (SSCF), and using high cell density fermentations with cell recycle, enhance the process productivity by removing sugar inhibition [53, 54].

## Conclusions

 In this work, a method of separating recalcitrant oligosaccharides from high solids-loading ACSH at a large scale was first developed. A series of acid hydrolysis and enzymatic hydrolysis with commercial cocktails were performed to understand the composition, recalcitrance and inhibitory effect of these oligosaccharides. As oligosaccharide accumulation in high solids-loading enzymatic hydrolysis was shown to be a universal problem for different pretreatment technologies (IL, DA and AFEX), this work provides a method to isolate and analyse oligosaccharides from different pretreated biomass, and facilitate studies to understand the mechanism behind their accumulation to develop strategies for better bio-refinery yield.

Using charcoal fractionation, we were able to separate and recover approximately 85 % of unhydrolysed oligosaccharides. The crude oligosaccharides were further fractionated based on their structural and molecular weight properties (neutral/acidic, different DP and molecular weights) using size exclusion chromatography. Acid and enzymatic hydrolysis showed that the low DP oligosaccharides became more digestible after being separated from monomeric sugars and degradation products in hydrolysate. High DP oligosaccharides; however, remained recalcitrant. Xylo-oligomers and arabino-oligomers were shown to have much higher recalcitrance compared to gluco-oligomers, giving clues about finding the potential unhydrolysed cross-linkages and targeting enzymes. Inhibition studies on commercial enzymes (Ctec2, Htec2 and MP) revealed that oligosaccharides have inhibitory effect on commercial enzymes. Low DP oligosaccharides were more inhibitory than high DP ones and the addition of monomeric sugars would further contribute to the inhibitory effect. Multifect pectinase possessed the highest digestibility over both neutral and acidic oligosaccharides, indicating the significance of adding appropriate accessory enzymes to work in synergy with cellulases and hemicellulases to fully convert the recalcitrant oligosaccharides into monomeric sugars. Overall, the mass distribution profile, recalcitrant study and enzyme inhibition study help us explore the complexity of recalcitrant oligosaccharides and to search for additional enzyme activities that are currently missing in commercial enzyme cocktails, thereby generating higher sugar conversion.

## Methods

### Biomass

Corn stover (CS) of Pioneer hybrid seed variety (33A14) was harvested in 2010 from Kramer farm in Wray (CO). Composition analysis was performed using the NREL protocol [55]. The composition of 2010 CS was 31.4 % glucan, 18.7 % xylan, 3.3 % arabinan, 0.0 % mannan, 1.2 % galactan, 2.2 % acetyl, 14.3 % lignin, 1.74 % protein and 13.39 % ash. Unless otherwise stated, ACS was used as is for enzymatic hydrolysis experiments without washing, conditioning, nutrient supplementation or detoxification.

### Enzymes

Cellic^®^ CTec2 (138 mg protein/mL, batch number VCNI 0001), a complex blend of cellulase, β-glucosidase and hemicellulase, and Cellic^®^ HTec2 (157 mg protein/mL, batch number VHN00001) were generously provided by Novozymes (Franklinton, NC, USA). Multifect Pectinase^®^ (72 mg protein/mL, batch number 4861295753) was a gift from DuPont Industrial Biosciences (Palo Alto, CA, USA). The protein concentrations of the enzymes were determined by estimating the protein (and subtracting the nonprotein nitrogen contribution) using the Kjeldahl nitrogen analysis method (AOAC Method 2001.11, Dairy One Cooperative Inc., Ithaca, NY, USA).

### Biomass pretreatment

DA pretreatment was performed at BESC (University of California, Riverside, CA, USA) at 160 °C for 20 min with 10 % w/w solid loading and 0.5 % w/w sulfuric acid using a 1-L Parr reactor with two stacked pitched blade impellers (Model 4525, Parr Instruments Company, Moline, IL, USA). It took 2 min for the reactor to reach 160 °C and another 2 min to bring the biomass temperature down to ambient conditions after pretreatment completion. The heating system was a 4-kW model SBL-2D fluidized sand bath (Techne, Princeton, NJ, USA). After the pretreatment, the residual solids were washed with water to remove acid and other degradation compounds produced during the process.

IL pretreatment was performed at JBEI (Berkeley, CA, USA) using 1-ethyl-3-methylimidazolium acetate, abbreviated as [C2mim][OAc], at 140 °C for 3 h using 15 % (wt/wt) loading of biomass to IL in a controlled-temperature oil bath using a sealed stirred vessel. It took 30 min for the reactor to reach 140 °C and 20 min to cool down to 60 °C. The residual IL was removed and pretreated biomass material was recovered with a series of water and ethanol washes.

AFEX pretreatment was performed at the GLBRC (Biomass Conversion Research Laboratory, MSU, Lansing, MI, USA). The conditions were 140 °C for 15 min at 60 % (wt/wt) moisture with 1:1 anhydrous ammonia to biomass loading in a bench-top stainless steel batch reactor (Parr Instruments Company) [48]. It took 30 min for the reactor to reach 140 °C and the ammonia was rapidly released, which immediately brought the biomass to room temperature. After the treatment, ammonia was removed by evaporation, leaving an essentially dry material. Hence AFEX is a dry to dry process, while IL and DA are dry to wet processes, as noted above.

### Chemicals

Celite 545 was purchased from EMD Millipore (Billerica, MA, USA). Activated charcoal (DARCO, 100 mesh particle), Avicel (PH-101), beechwood xylan and all other chemicals were purchased from Sigma–Aldrich (St. Louis, MO, USA).

### Compositional analysis

Extractive-based compositional analyses of the samples were performed according to the NREL LAPs: Preparation of Samples for Compositional Analysis (NREL/TP-510-42620) [56] and Determination of Structural Carbohydrates and Lignin in Biomass (NREL/TP-510-42618) [55]. The biomass was extracted with water and ethanol prior to the acid hydrolysis step.

### Oligosaccharide analysis

Oligomeric sugar analysis was conducted on the hydrolysate liquid streams using an autoclave-based acid hydrolysis method at a 2-mL scale. Hydrolysate samples were mixed with 69.7 μL of 72 % sulfuric acid in 10 mL screw-cap culture tubes and incubated in a 121 °C bench-top hot plate for 1 h, cooled on ice and filtered into HPLC vials. The concentration of oligomeric sugar was determined by subtracting the monomeric sugar concentration of the non-hydrolysed samples from the total sugar concentration of the acid hydrolysed samples. Sugar degradation was accounted for by running the appropriate sugar recovery standards along with the samples during acid hydrolysis.

### High solids-loading enzymatic hydrolysis

High solids-loading 25 % (w/w) (approximately to 8 % glucan loading) ACSH was prepared as starting material for the large-scale production of oligosaccharides. Enzymatic hydrolysis of ACS was performed using a commercial enzymes mixture including Cellic^®^ Ctec2 10 mg protein/g glucan (in pretreated biomass), Htec2 (Novozymes, Franklinton, NC), 5 mg protein/g glucan and Multifect Pectinase (Genencor Inc, USA), 5 mg protein/g glucan. Enzymatic hydrolysis was carried out in a 5-L bioreactor with 3 L working volume at pH 4.8, 50 °C and 250 rpm. After 96 h of hydrolysis, the hydrolysate was harvested by centrifugation at 6000 rpm for 30 min and then 14,000 rpm for 30 min to remove unhydrolysed solids. Hydrolysate was then sterile filtered through a 0.22-um filter cup. The filtered hydrolysate was stored at 4 °C in a sterile bottle prior to charcoal fractionation (described below). Samples obtained from compositional analysis were subjected to HPLC using Bio-Rad Aminex HPX-87H column to determine sugar concentrations as described below.

### Micro-plate based enzyme recalcitrance study

Micro-plate based enzymatic hydrolysis was performed in 0.5 mL reaction volumes with commercial enzymes (Ctec2:Htec2:MP) in 1:1:1 ratio for 24 h hydrolysis at 50 °C and pH 4.8. Maximum sugar concentration per well was 3 g/L while the minimum sugar concentration per well was 0.2 g/L (determined by acid hydrolysis). Enzyme loading is 60 μg/well, i.e., 20–300 mg/g glucan.

### Charcoal fractionation of oligosaccharides

An activated charcoal and Celite mixture was used to extract oligosaccharides (affinity and polarity based extraction) from the hydrolysate prepared above [50, 51]. Activated charcoal (250 g) and Celite powder (250 g) were thoroughly mixed and packed in a 1-L sintered glass filter funnel connected to a 2-L vacuum flask. The charcoal–Celite mix was first preconditioned in 100 % acetonitrile overnight to clean and activate the charcoal mixture. After the charcoal matrix being soaked in acetonitrile overnight, 6–8 L of water was added with vacuum filtration to prepare the matrix for hydrolysate incubation. Before the last of this water drained out, 500 mL of hydrolysate was added the charcoal matrix and left overnight to allow adsorption of oligosaccharides to the charcoal matrix. After incubation, another 5 L of water followed by 2 L of 5 % acetonitrile (v/w) was used to wash the un-adsorbed and loosely bound materials. Following this wash step, acetonitrile (ACN) and formic acid (FA) were used as eluents to desorb the oligosaccharides from the charcoal matrix. 3 L of 50 % acetonitrile (v/w) were used to separate neutral oligosaccharides, followed by 2 L of 50 % acetonitrile with 1 % formic acid (v/w) to separate the acidic oligosaccharides. After collecting both the neutral and acidic fractions separately, 2 L of 60 % methanol (v/w) was used to wash the charcoal matrix. Vacuum can be applied to fasten the fractionation process but longer interaction times between the hydrolysate and elution solvent (acetonitrile and formic acid) with charcoal matrix are beneficial for higher recovery. The charcoal matrix should be submerged in solvent (not allowed to dry out) throughout the process.

### Desalting and concentration of crude oligosaccharide fractions

Crude oligosaccharides from charcoal fractionations were concentrated using a SpeedVac concentrator (SC210P1-115, Thermo Scientific) before SEC fractionation. Acetonitrile was removed first and the oligosaccharides were re-suspended with water (50 mL of neutral fractions/55 mL of formic acid fractions). Ammonium hydroxide was used to neutralise formic acid (pH adjusted to 5.5) in acidic samples and volatile ammonium formate was removed with water addition using same repeated SpeedVac cycles. The desalting procedure was repeated at least five times to remove most of the salts.

### Micro-plate based enzyme inhibition study

Micro-plate enzyme activity assay experiments were performed at enzyme loading of 10 mg/g substrate, i.e., 62.5 μg enzyme per well, for 24-h hydrolysis at 50 °C, 10 rpm and pH 4.8 (adjusted by adding 1 M citrate buffer pH 4.3). Reaction volume for each well is 500 μL. Pure substrates (Avicel and beechwood xylan) were added in 1.25 % solids loading, i.e., 6.25 mg per well. Oligosaccharide concentration was at 10 g/L; monomeric sugars were at 20 g/L glucose, 10 g/L xylose. Avicel was tested using Ctec2; beechwood xylan was treated with Htec2 and MP individually, and another mixture of Htec2 and MP in 1:1 mass ratio.

### Analytical method

Glucose, xylose and arabinose concentrations were analysed using a Shimadzu HPLC system equipped with a Bio-Rad Aminex HPX-87H column equipped with automatic sampler, column heater, isocratic pump and refractive index detector (RID). The column was maintained at 50 °C and eluted with 5 mM H_2_SO_4_ in water at 0.6 mL/min flowrate. Monomeric sugars were identified and quantified by comparison to authentic standards using a five-point calibration curve.

#### Fractionation based on size exclusion chromatography (SEC) column

The concentrated neutral and acidic samples were fractionated using size exclusion chromatography (FPLC Amersham-Biosciences, Akta system 890) with an XK 260 × 1000 mm column (GE Healthcare) packed with P2 gel (200–400 mesh, separation range of 100–1800 MW, Biorad Laboratories, Hercules, CA, USA). The instrument was equipped with both Ultra-Violet and conductivity detectors (GE Healthcare). Water was used as eluent solvent. A flow rate of 1.0 mL/min was used and a 5-mL sample was injected for each run. 5 mL of concentrated neutral fractions (containing 670 mg oligosaccharides) and acidic fractions (containing 140 mg oligosaccharides) were injected into the SEC column after a 500-mL void volume. 90 fractions (A1-12, B1-12, C1-12, D1-12, E1-12, F1-12, G1-12, H1-6, 10 mL in each tube) were collected. Fractions in the same row were pooled and lyophilized into dry sample A–H by a freeze-dryer (FreeZone Plus 6 Liter Cascade Console Freeze Dry System, LABCONCO).

## Abbreviations

AFEX: ammonia fibre expansion
CS: corn stover
ACS: AFEX™ pretreated corn stover
ACSH: AFEX™ pretreated corn stover hydrolysate
SEC: size exclusion chromatography
DP: degree of polymerization
MP: multifect pectinase

## Footnotes

1 ^a^TM—AFEX is a trademark of MBI International, Lansing, Michigan.
